# Career outcomes of PhD holders in Africa: Protocol for a scoping review

**DOI:** 10.1371/journal.pone.0350289

**Published:** 2026-08-04

**Authors:** Aimee Haley, Alebachew Kemisso Haybano, Gun-Britt Wärvik

**Affiliations:** 1 Department of Education and Special Education, University of Gothenburg, Gothenburg, Sweden; 2 Centre for Comparative Education and Policy Studies, Addis Ababa University, Addis Ababa, Ethiopia; Higher Education Partnership / Erasmus University Rotterdam, ETHIOPIA

## Abstract

The purpose of this paper is to outline a protocol for a scoping review that will map current research on PhD holders’ career outcomes in Africa. The aim of the scoping review is threefold. First, it seeks to examine the predominant conceptual and theoretical frameworks, as well as the methodological approaches, used in existing research. Second, the review aims to assess the extent to which academic and non-academic careers are addressed within the current literature. Lastly, the scoping review intends to identify the systemic, institutional, and individual-level conditions that contribute to shaping research careers. Four electronic journal databases were searched for peer-reviewed articles in English that focused on the African context: Web of Science, Scopus, the Sabinet Social Sciences and Humanities Journal Database, and Education Research Complete. No restrictions were placed on publication year, with searches including studies published up to 2025. The abstracts and titles of the sources resulting from this search will be screened against the established eligibility criteria, and data will be extracted and analyzed from those sources that meet the inclusion criteria. The findings from this scoping review will be useful for research leaders, as well as those responsible for the professional development of researchers who seek to establish career development initiatives. This review will also point out research gaps in career-related literature on PhD holders in Africa.

## Introduction

A common belief is that knowledge development and innovation in national contexts depend on high-quality research that is both socially relevant and impactful [1]. Consequently, individuals who have been trained to conduct research, such as PhD holders, play an integral role in national economic development and poverty reduction [2]. However, globally, early-career academics increasingly encounter unstable conditions that limit their ability to build sustainable careers within academia. Insecure employment situations, closed or informal hiring practices, and weak support structures leave many new PhD holders in uncertain career trajectories [3]. These dynamics are not uniform, but they reflect broader pressures on higher education systems under conditions of expansion and resource constraints. In many African contexts, these pressures are intensified by chronic underfunding and structural inequalities, resulting in considerable development challenges [4]. Although doctoral training has expanded on the continent, a plethora of research indicates that African researchers with doctoral degrees struggle to contribute to global knowledge production and progress in research careers, both within and outside of academia [5–7]. Moreover, the problem of limited collaboration between the higher education sector and industry in Africa, despite many PhD holders working within industry, risks further constraining the broader development impact of advanced research training [8]. Even though these challenges are recognized, there remains limited systematic evidence of the conditions that affect the research career outcomes of PhD holders in African countries.

Several systematic and scoping literature reviews have already been published about the career outcomes of PhD holders [9–12]. Noticeably absent from these existing reviews is research on the African context, and when publications about the African context have been included, they have been few and not the focus of the review. This is precisely where this proposed scoping review will contribute.

### Aim

This paper outlines a protocol for a scoping review of the existing literature on the career outcomes of PhD holders in Africa. The protocol describes the research design of the planned scoping review, including materials and methods, as well as its anticipated contributions and limitations. The objectives of this scoping review are: (1) To map the conceptual and theoretical frameworks, as well as the methodological approaches, that have been used by researchers to investigate the careers of PhD holders in Africa; (2) To describe the prevalence of research on academic and non-academic careers; and (3) To identify the conditions that shape research career outcomes. In particular, the review will identify what systemic-, institutional-, and individual-level conditions contribute to shaping research careers. Attention to conceptual and theoretical frameworks is necessary since they shape the aspects of careers that researchers examine and how relationships between influencing factors and career outcomes are interpreted. The research questions guiding this scoping review are:

- What conceptual and theoretical frameworks, as well as methodological approaches, have been used to study the career outcomes of PhD holders in Africa?
- What types of career outcomes (e.g., employment sectors) of PhD holders in Africa are most researched?
- How do broader systems, institutional environments, and individual circumstances contribute to shaping research careers in Africa?

## Materials and methods

The purposes of a scoping review include examining the extent, range, and nature of research, summarizing existing findings, identifying research gaps, and exploring whether a full systematic review is warranted. Unlike systematic reviews, scoping reviews do not assess research quality and often encompass a broader range of research [13]. As the aim is to examine existing research on PhD holders’ careers in Africa without assessing methodological quality, a scoping review is appropriate. The JBI guidelines for scoping reviews also advise against quality assessment [14]. This approach is particularly relevant given the interdisciplinary nature of the topic, where standards of sound methodology vary across disciplines.

Since this study will review already published works, ethical clearance is not required. Nevertheless, transparency and integrity in the selection process, avoidance of bias, and due recognition of original authors and research participants remain essential ethical responsibilities [15]. As researchers, reflexivity regarding assumptions, values, and positionality will be maintained, alongside consideration of how these might shape the understanding and interpretation of the data.

The scoping review process began in March 2025 with preliminary database searches and the development of a protocol outline in May 2025. The search strategy has since been refined and updated to incorporate additional terms. Screening and data extraction are ongoing. Analysis and write-up are expected in mid-2026, with the aim of submitting the full scoping review for publication by the end of 2026.

To uphold methodological rigor, this scoping review follows guidelines from the Joanna Briggs Institute (JBI) [14]. Accordingly, this section outlines the eligibility criteria, search strategy, evidence screening and selection, data extraction, and data analysis and presentation of results.

### Eligibility criteria

In literature reviews, a clear and comprehensive outline of inclusion and exclusion criteria is crucial for guiding the review process. The criteria used in this scoping review were formulated following the population-concept-context (PCC) framework, which is herein described.

- Population – This review will focus on research about PhD or doctorate holders in Africa. Sources that focus solely on master’s degree holders, doctoral students, or medical doctors will be excluded, though sources that include these groups, as well as PhD holders, will be included in this scoping review.
- Concept – In this review, the concept in focus is “career outcomes”. Career outcomes may pertain to university and non-university sectors and include all career stages.
- Context – The context or setting is Africa. Sources that focus on career outcomes in African countries will be included, while sources that focus on other contexts will be excluded.

While the PCC framework makes clear the most important eligibility aspects for selecting sources, additional parameters relating to source type and language were applied. Only sources based on original peer-reviewed research will be included, while others will be excluded (e.g., conceptual papers, literature review studies, and editorials).

### Search strategy

A clear search strategy is an important aspect in the process of conducting a scoping review. Based on the nature of the research questions, the search will be limited to scientific databases. As the topic crosses all scientific fields, two interdisciplinary databases were selected: Web of Science and Scopus. Education Research Complete was also included to catch studies that may not be fully represented in interdisciplinary databases, such as literature linking doctoral education to career outcomes. A country/region filter was applied to select all African countries. Truncation (“*”) and Boolean operators (AND/OR) were used to search the titles, abstracts, and keywords of sources in the databases. The search terms used were: (doctora* OR PhD AND career*) and (doctora* OR PhD and profess* OR employ*), and the selection of these search terms and parameters is an operationalization of the criteria that were described in the PCC framework.

As it is well known that there are geographical disparities in the coverage of journals in these two databases and journals published in sub-Saharan Africa are the most underrepresented [16], it was necessary to search an African-specific database called Sabinet Social Sciences and Humanities Journal Database. The same Boolean operators were used to search this database. For all databases, search parameters were set to sources in English. No restrictions were placed on publication year, with searches including studies published up to 2025. Using these parameters, the search yielded 1,168 sources from Web of Science, 712 from Scopus, 53 from Education Research Complete, and a further 1,043 from Sabinet Social Sciences and Humanities Journal Database.

### Evidence screening and selection

The sources identified through this process will be compiled in the reference management software program called EndNote, and duplicates will be removed manually. The remaining sources will be transferred to the Rayyan collaboration and research tool [17]. Rayyan is a web application that will be used by the authors to collaborate in reviewing source titles and abstracts against the eligibility criteria. The first author and the second author will conduct an independent blind review of all sources and will decide whether they should be reviewed in full. The third author will serve as the tie breaker for the disagreements that arose between the two. Thereafter, the remaining sources will be divided equally between the three authors, where they will be read in full, and data will be extracted.

### Data extraction

Data from the included sources will be added to a data extraction form in Microsoft Excel. The data extraction form was jointly designed by all authors and includes several categories on which to collect data from the sources (see S1 File). Before proceeding to review all included sources in full, the authors will conduct a trial run of the form with a few selected sources. Then, any clarifications and adjustments to the form can be made before commencing the fulltext review. The categories on the form include bibliometric aspects of the sources (e.g., the authors and publication year), aspects related to the PCC framework (e.g., national context and conditions affecting career outcomes of PhD holders), and aspects related to the conceptual and theoretical frameworks and methodological approaches employed in the studies. Any sources identified in this phase as not meeting the inclusion criteria will be noted and excluded from the scoping review. The reasons for these exclusions will be recorded in a flow diagram, which will be adapted from the PRISMA guidelines and extension for scoping reviews [18].

### Data analysis and presentation of results

**Fig 1:**
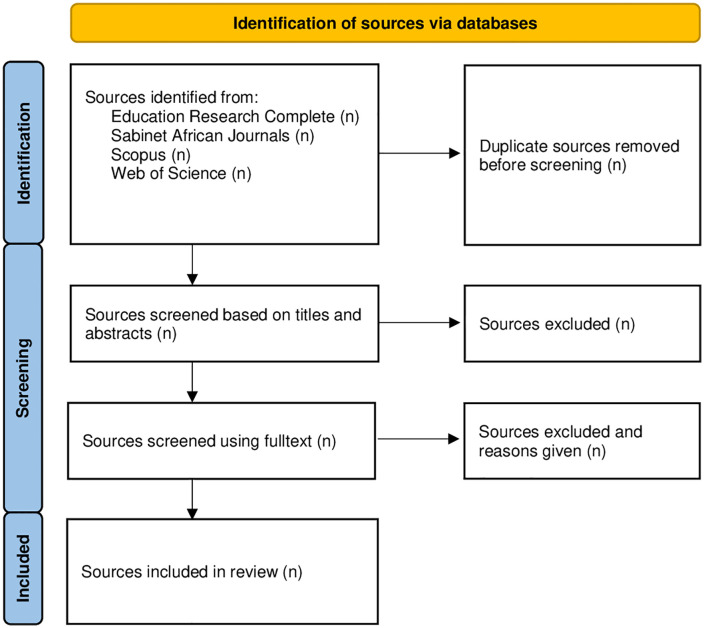
Flow diagram of the source search and selection process.

In the presentation of results, the study will begin by explaining the process for selecting sources for inclusion in this literature review and by showing this process in the flow diagram. See Fig 1 for an example of the flow diagram, which illustrates the source search and selection process. The results will then be presented in response to the three research questions as frequencies and percentages, using tables or charts, and summarized in text. Lastly, a qualitative content analysis of the systemic, institutional, and individual conditions shaping research careers will also be presented, which corresponds to the third research question.

## Contributions and limitations

This scoping review will provide the most recent and comprehensive account of the career outcomes of PhD holders in Africa. The review will contribute systematic evidence on conditions at the individual, institutional, and systemic levels that relate to research career outcomes. Such knowledge is needed to identify weaknesses and contextual challenges that may need to be considered by African universities when developing research career development initiatives. By systematically mapping the conditions, the review will also identify areas where further research and attention are needed. In addition, the review will highlight the conceptual and theoretical frameworks, as well as the methodological approaches, most commonly used in existing research and point to research designs that could be considered in future studies. The findings will be published in a scholarly journal and will serve as a foundation for a research project recently funded by the Swedish Research Council [19].

Like all research studies, this scoping review has certain limitations. Since the search is limited to four journal databases and English-language publications, some relevant sources, especially from African countries where the dominant language in academia is not English (e.g., Mozambique), may be missed. The decision not to assess the quality of included sources is, on one hand, inclusive and allows for a wider range of perspectives, findings, and contexts. On the other hand, this may result in the inclusion of sources with methodological limitations, potentially affecting the strength of the conclusions drawn. Despite these limitations, the findings will be valuable for research leaders and those responsible for developing researchers’ career development initiatives. This review will also identify gaps in the literature on the careers of PhD holders in Africa and will be useful for researchers in this area.

## Supporting information

S1 File Data extraction form. (PDF)

S1 Table PRISMA-P checklist. (PDF)
