# Supplementary material for: Career outcomes of PhD holders in Africa: Protocol for a scoping review
Source: PLoS One. 2026 Aug 4;21(8):e0350289. doi: 10.1371/journal.pone.0350289 (PMC13436724; doi:10.1371/journal.pone.0350289)
Supplement: Supplementary file 1 [file pone.0350289.s001.pdf]

Authors and publication year	Exclude? (If yes, give the reason)	Research aim(s)	Research problem	Focal country	Participants' career field	Theory / Concepts	Method	Research design (e.g., longitudinal, experimental, cross-sectional)	Number of participants	Data collection instrument available? (yes/no)	Main finding	Career type	Conditions that affect research career outcomes		
													System	Instituion	Individual
